# Commentary: Probing the Neural Mechanisms for Distractor Filtering and Their History-Contingent Modulation by Means of TMS

**DOI:** 10.3389/fnins.2020.00365

**Published:** 2020-04-15

**Authors:** Dominic M. D. Tran

**Affiliations:** School of Psychology, The University of Sydney, Camperdown, NSW, Australia

**Keywords:** distractor filtering, attention, brain stimulation, transcranial magnetic stimulation (TMS), frontal eye field

The ability to suppress irrelevant but attention grabbing stimuli in our environment is critical for maintaining goal-directed control during many daily activities, such as avoiding junk foods while dieting or ignoring distractions while driving. Although we know much about the properties of environmental cues that make them susceptible to attentional capture (e.g., bright lights, loud sounds, fast movements), we know far less about how to disengage from such distractions, particularly the neural mechanisms involved in these processes.

In a recent paper by Lega et al. (2019), the authors examined whether non-invasive brain stimulation, using transcranial magnetic stimulation (TMS), could be used to modulate distractor filtering when applied over the dorsal frontoparietal attentional networks, specifically the frontal eye field (FEF) and the intraparietal sulcus (IPS). The authors used a visual search task in which participants had to identify a target within a four-item array. Critically, on half of the trials, the array also included a salient distractor in a pop-out color (e.g., displayed in red while all other items in the array, including the target, were displayed in green). On distractor-*absent* trials, participants were fast to find the target, and as expected, on distractor-*present* trials, participants were slow; their attention was effectively captured by the pop-out color, slowing their search and identification of the target in the array.

Interestingly, the authors showed that delivering triple-pulse 10 Hz TMS at 100% of resting motor threshold to the right FEF 100 ms after the presentation of the search array reduced the slowing of reaction times (RTs) on distractor-present trials compared to stimulation over a control (sham) location. Notably, this reduction to the RT interference effect on distractor-present trials following FEF stimulation was specific to the right hemisphere and did not have the same effect over the left hemisphere. Moreover, right IPS stimulation generated a similar reduction in RT slowing on distractor-present trials compared to the sham stimulation, but this difference was not statistically significant and numerically smaller than the difference produced by right FEF stimulation. Although no discussion was provided about the TMS parameters selected and how this may affect the selectivity of the effect, the results indicate that targeting the right FEF produced the largest modulatory effects on distractor processing. This is a fascinating and highly relevant finding because it suggests that non-invasive brain stimulation may have the potential to train or improve attentional control over task-irrelevant, salient distractors.

Importantly, the benefit to RT through reduced distractor interference following right FEF stimulation can be interpreted in two ways. One explanation is that stimulation of the right FEF improved distractor filtering processes by activating neural circuits involved in attention regulation, allowing for more successful disengagement and control of task-irrelevant information. A second explanation is that the stimulation impaired attention biasing, or pop-out detection, processes by disrupting neural circuits responsible for attentional capture from salient visual features. Lega et al. (2019) favor the enhancement of distractor filtering explanation and rule out the disruption of attentional capture explanation, but this interpretation warrants further discussion.

Lega et al. (2019) argued that if stimulation of the right FEF disrupted attentional capture by impairing saliency computation, performance on distractor-absent trials should also be impacted. Specifically, the authors suggest that target detection in and of itself also involves some saliency computation and that if this process was disrupted, RT even on distractor-absent trials should be slowed by TMS over the right FEF relative to the sham location. However, we know that color change, which was the feature change for the distractor item, is more salient than shape change, which was the feature change for the target item, in a visual search paradigm (Theeuwes, 1991). That is, the feature difference between green and red stimuli is larger than the feature difference between a triangle and a rotated triangle. Thus, it is possible that TMS may impair saliency computation for highly distracting feature changes such as pop-out color while leaving intact the ability to detect more subtle shape changes in an array that may not immediately pop-out.

A second reason Lega et al. (2019) favor the distractor filtering account is that they claim it provides a simpler explanation of their cross-trial contingency findings. In addition to showing that right FEF stimulation facilitated target detection on distractor-present trials, they also found that target detection was affected by the previous trial type. If the previous trial was also a distractor-present trial, then the RT cost of having a distractor present on the current trial was reduced compared to instances in which the previous trial was a distractor-absent trial. Put simply, participants were better at finding the target alongside a distractor if they had just done so on the previous trial. These cross-trial contingency effects in pop-out visual search have been previously documented in the literature [e.g., (Geyer et al., 2008); similar sequential effects have also been observed with the Eriksen flanker task, (Gratton et al., 1992), and the Simon task, (Hommel et al., 2004)] but not in combination with TMS. Lega and colleagues additionally demonstrated that right FEF stimulation eliminated the cross-trial contingency effect. That is, performance was just as good on the present trial whether or not there was a distractor present on the previous trial.

Lega et al. (2019) posit that the elimination of the cross-trial contingency effect by TMS stimulation is more easily explained by the enhancement of distractor filtering account than the suppression of attentional capture account. However, both explanations are plausible with respect to their results. For example, if right FEF stimulation disrupted the neural circuits responsible for attentional capture by salient visual features, this will result in both the previous and current distractor-present trials as being essentially comparable to distractor-absent trials, thereby eliminating the cross-trial contingency effect. Independently of which explanation is more parsimonious, both explanations of the cross-trial contingency TMS effect remain purely speculative since TMS was delivered on every trial. Any cross-trial contingency TMS effects cannot be isolated to whether performance on the current trial is being affected by stimulation that occurred on the current or previous trial. Future studies investigating cross-trial contingency effects in combination with TMS over the right FEF should include non-TMS trials to disentangle the effect of stimulation on the current trial vs. effects persisting from stimulation on the previous trial.

More experimental work is needed to test the competing hypotheses of distractor filtering and attentional capture. Once these mechanisms have been teased apart, interesting avenues can be explored as the current findings on TMS modulation of distractor processing are of wide interest and relevance. For example, the findings have implications for recent work showing that rewarding stimuli can involuntarily capture attention in a visual search paradigm even when they directly conflict with current goals [e.g., (Anderson et al., 2011; Le Pelley et al., 2015); see (Anderson, 2016) for review]. Specifically, learning that a stimulus is predictive of a high value reward can increase the attentional capture by that stimulus, even if looking at that stimulus instead of a target omits the delivery of that high value reward. Interestingly, this counterproductive “value”-modulated capture by rewarding stimuli resembles the attentional biases toward drug-associated stimuli seen in addiction pathologies, and indeed the two phenomena have been shown to be related (Anderson et al., 2013; Albertella et al., 2017).

Whereas feature-based attentional capture works through the physical salience of the distractors, value-modulated attentional capture works through their reward salience (Della Libera and Chelazzi, 2009; Hickey et al., 2010; Hickey and van Zoest, 2012). These differing capture processes have been shown to involve common and unique neural pathways (Anderson et al., 2014). It is perhaps unsurprising that processing and filtering distractors in both cases share overlapping mechanisms, especially considering they are both visual capture paradigms and that value-modulated attentional capture often piggybacks off the physical salience of the distractors. Having common mechanisms could mean that the findings by Lega et al. (2019) would offer a novel avenue for addiction treatment by providing a neural basis for reducing the attentional reactivity to drug-associated stimuli. Future research can test the efficacy of this possibility by using a value-modulated attentional capture paradigm in combination with TMS targeted over the right FEF [possibly even the inferior frontal gyrus or middle frontal gyrus; see (Anderson et al., 2014)]. However, before these potentially exciting clinical implications can be explored, the reliability and robustness of the current findings need to be demonstrated. In doing so, bettering our understanding of the neural circuits involved in distractor processing may lead the way in developing non-invasive brain stimulation therapies for improving attention regulation and control in people with conditions involving attentional deficits.

## Author Contributions

DT wrote the manuscript.

### Conflict of Interest

The author declares that the research was conducted in the absence of any commercial or financial relationships that could be construed as a potential conflict of interest.
